# Sporadic Human Cryptosporidiosis Caused by *Cryptosporidium cuniculus*, United Kingdom, 2007–2008

**DOI:** 10.3201/eid1703.100410

**Published:** 2011-03

**Authors:** Rachel M. Chalmers, Kristin Elwin, Stephen J. Hadfield, Guy Robinson

**Affiliations:** Author affiliations: Public Health Wales Microbiology–Singleton Hospital, Swansea, UK

**Keywords:** Cryptosporidium cuniculus, human cryptosporidiosis, prevalence, epidemiology, GP60, parasites, United Kingdom, dispatch

## Abstract

To investigate sporadic human cryptosporidiosis trends in the United Kingdom, we tested 3,030 *Cryptosporidium* spp.–positive fecal samples, submitted for routine typing in 2007–2008, for *C. cuniculus*. *C. cuniculus* prevalence was 1.2%; cases were mostly indigenous and occurred across all age groups. Most occurred during August–October and may be linked to exposure opportunities.

The protozoan parasites *Cryptosporidium* spp. are major causes of gastrointestinal disease worldwide. Most cases in the United Kingdom are caused by *C. parvum* or *C. hominis*; rare infections with other species and genotypes include *C. meleagridis*, *C. felis*, *C. canis*, *C. ubiquitum*, *C. hominis* monkey, skunk, horse, and rabbit (*1**–**3*). In summer 2008, the rabbit genotype caused a waterborne outbreak in drinking water (*4*). Previously, only 1 human infection, also identified in the United Kingdom, was known (*1*), although routine typing based on *RsaI* restriction fragment-length polymorphisms (RFLPs) within the *Cryptosporidium* oocyst wall protein (COWP) gene (*2*) does not differentiate the rabbit genotype from *C. hominis* (*4*). After phylogenetic and biologic investigations, the rabbit genotype has been renamed *C. cuniculus* (*5*). However, information is lacking about the occurrence and epidemiology of animal and human infections outside the outbreak, which mainly involved adult females (*5**–**7*). To investigate trends in humans, we conducted enhanced testing of *Cryptosporidium* spp.–positive fecal samples from patients with sporadic diarrhea (submitted for routine typing during 2007–2008) with the purpose of identifying and characterizing *C. cuniculus*.

## The Study

Archived DNA from all samples received for typing during January 2007–December 2008 that showed *C. hominis* COWP PCR-RFLP RsaI profiles were retested by single-round small subunit (SSU) rRNA PCR-RFLP using *SspI*, which generates a pattern unique to *C. cuniculus* (*4*). An exception occurred during the outbreak period (July and August 2008) when all *Cryptosporidium* spp.–positive stool samples were tested by a pan-genus nested PCR specific for the SSU rRNA gene and products digested with *SspI* and *VspI* (*4*). Although differentiating more species/genotypes than the COWP PCR-RFLP (*7*), this assay is unsustainable for typing large numbers of samples on a routine basis. *C. cuniculus* was confirmed by sequencing ≈830 bp of the SSU rRNA gene and ≈850 bp of the 60-kDa glycoprotein (GP60) gene by using nested PCR protocols (*4*).

Data were analyzed in Epi Info version 6 (Centers for Disease Control and Prevention Atlanta, GA, USA). Incomplete data for Northern Ireland were excluded, but no *C. cuniculus* cases were identified there. Case-patients with sporadic *C. cuniculus*, *C. parvum*, and *C. hominis* infections were compared by age using the Mann-Whitney 2-sample test, by sex using the Mantel-Haenszel version of the χ^2^ test, by month of specimen submission, and by Government Office Region (England and Wales) or Health Board (Scotland) of the primary diagnostic laboratory.

In total, 37 (1.2%) of 3,030 infections were caused by *C. cuniculus*: 23 in 2007 and 14 in 2008 (Table A1). Twenty-five were in patients from England and Wales, and 12 patients were from Scotland. Other cryptosporidia detected were *C. parvum* (n = 1,506, 49.7%), *C. hominis* (n = 1,383, 45.6%), *C. meleagridis* (n = 26), *C. felis* (n = 8), cervine genotype (n = 8), co-infection *C. hominis* and *C. parvum* (n = 5), novel or unidentified genotypes (n = 5), and *C. hominis* monkey genotype (n = 1); 88 did not amplify with the PCR primers. Substitution of routine typing with the SSU rRNA nested PCR-RFLP during the outbreak did not increase the number of “unusual” cryptosporidia, apart from *C. cuniculus*, indicating the routine COWP PCR-RFLP is otherwise appropriate for typing for epidemiologic purposes in the United Kingdom.

**Figure 1:**
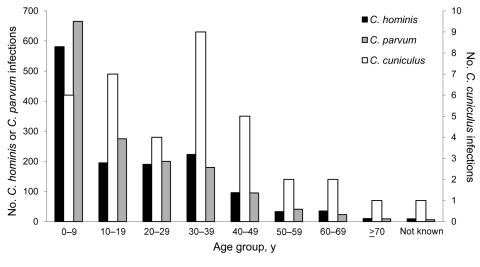
Age distribution of patients with sporadic cases of *Cryptosporidium cuniculus*, *C. hominis*, and *C. parvum* infection in England, Wales, and Scotland, 2007–2008.

The age range of patients with sporadic *C. cuniculus* infection was 1–74 years (mean 29 years; median 31 years), significantly older than *C. hominis* case-patients (range 0–83 years; mean 19 years; median 13 years) (Mann-Whitney 2-sample test value = 11.12, df = 1, p = 0.0009) and *C. parvum* case-patients (range 0–86 years; mean 17 years; median 29 years) (Mann-Whitney 2-sample test value = 15.24, df = 1, p = 0.00009) (Figure 1). The sex distribution was 14 (37%) female and 22 (58%) male patients, with the sex of 1 patient not known, compared with sporadic *C. parvum* (781 [51.9%] female) and *C. hominis* (736 [53.2%] female) cases, although the difference was not significant (χ^2^ = 4.01, df = 2, p = 0.13).

**Figure 2:**
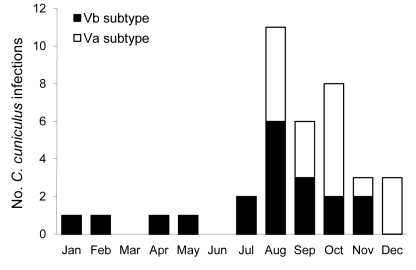
Monthly distribution of sporadic cases of *Cryptosporidium cuniculus*, *C. hominis*, and *C. parvum* infection in England, Wales, and Scotland, 2007–2008.

More *C. cuniculus* cases were detected in the late summer and autumn than in the winter and spring, similar to infections with *C. hominis* but not *C. parvum* (Figure 2). Three *C. cuniculus* cases were identified in the East Midlands, the outbreak affected region, but none were found in Northamptonshire, the outbreak-affected area. Most cases (24%) were in the Eastern region of England. Two case-patients had traveled outside the UK, one to Spain and the other destination not known, during the incubation period.

Occupational and environmental exposure data were available for 14 *C. cuniculus* case-patients from patient questionnaires administered by local Environmental Health Departments. No occupational risks were linked to rabbits. One patient (a 9-year-old boy) reported direct contact with rabbits (a pet), and 2 patients had potential environmental contact (a 63-year-old woman played golf, and a 36-year-old man sat on grass during a walking holiday). Two case-patients reported diarrhea in other residential contacts. None of the patients reported links with (lived in, had visited or received visitors from) the outbreak-affected area. Available clinical details were insufficient for all cryptosporidiosis case-patients for comparative purposes. One *C. cuniculus* case-patient was an immunosuppressed child who had received a kidney transplant.

Two GP60 subtype families, Va (n = 18) and Vb (n = 19), were detected in sporadic *C. cuniculus* isolates, linked to patient sex; 10/14 (71.4%) female patients had Va subtype, compared with 7/22 (31.8%) male patients (χ^2^ = 5.24, df = 1, p = 0.022). No significant difference in age or regional distribution was found, but cases with Va occurred only in August through December, while Vb cases occurred all year but mostly in August (Figure 2).

Representative sequences have been deposited into GenBank: GU971631–GU971650 (GP60) and GU971628–GU971630 (SSU DNA). The latter are identical to those deposited previously (EU437413, FJ262724–FJ262726) (*1**,**4*) and are *C. cuniculus* (*5*).

## Conclusions

*C. cuniculus* was first identified as a human pathogen during a waterborne outbreak, and its epidemiology has now been described for sporadic cases in the United Kingdom. Although the numbers are small, and the data need to be interpreted with caution, it was the third most commonly identified *Cryptosporidium* species in patients with diarrhea during the study period, after *C. parvum* and *C. hominis*. All *C. cuniculus* isolates identified by PCR-RFLP were confirmed by sequence analysis, indicating the reliability of the test algorithm used, although the development of specific probes will enhance testing capability. All ages were infected with little age delimitation <50 years, after which numbers declined. Contrast this to *C. parvum* and *C. hominis*, which are both linked to young age. *C. cuniculus* distribution is seasonal, peaking in August through November, and differences in seasonal distribution of GP60 subtypes were marked.

Rabbits are the natural hosts for *C. cuniculus* (*5*), and seasonal distribution and variation in humans may reflect rabbit breeding seasons and infections, although good epidemiologic studies of *Cryptosporidium* spp. in wild rabbits are lacking (*7*). Studies of farmed and wild rabbits so far indicate that GP60 subtype Vb predominates (*7**–**9*), although the outbreak was caused by Va (*4*). Unlike with *C. hominis*, seasonal distribution of cases was not linked to foreign travel. The *C. cuniculus* outbreak had occurred in July when few sporadic cases were detected. The distribution of sporadic cases was the opposite of the outbreak in which more case-patients were female (*6*). The association between GP60 subtypes and patient sex is intriguing. A small number of case-patients had possible exposure risks, although this requires further investigation. The only known hosts of *C. cuniculus* are humans and European rabbits (*Oryctolagus cuniculus*) (*5**,**7**–**9*), and until population-based studies of rabbits are undertaken, the risks cannot be fully evaluated, nor the human epidemiology fully explained.

## Appendix

**Table A1:** Demographic information on patients with sporadic *Cryptosporidium* spp. infection, by species, England, Wales, and Scotland, 2007–2008

Characteristic	No. (%) patients			
	*C. cuniculus* infection, n = 37	*C. hominis* infection, n = 1,383		*C. parvum* infection, n = 1,506
Year				
2007	23 (62.2)	461 (33.3)		716 (47.5)
2008	14 (37.8)	922 (66.7)		790 (52.5)
Age, y				
0–9	6 (16.2)	581 (42.0)		665 (44.2)
10–19	7 (18.9)	195 (14.1)		275 (18.3)
20–29	4 (10.8)	190 (13.7)		200 (13.3)
30–39	9 (24.3)	223 (16.1)		180 (12.0)
40–49	5 (13.5)	96 (6.9)		95 (6.3)
50–59	2 (5.4)	33 (2.4)		41 (2.7)
60–69	2 (5.4)	35 (2.5)		23 (1.5)
>70	1 (2.7)	10 (0.7)		9 (0.6)
Sex				
M	22 (59.5)	616 (44.5)		704 (46.7)
F	14 (37.8)	736 (53.2)		781 (51.9)
Month of diagnosis				
January	1 (2.7)	52 (3.8)		53 (3.5)
February	1 (2.7)	34 (2.5)		38 (2.5)
March	0	20 (1.4)		78 (5.2)
April	1 (2.7)	26 (1.9)		178 (11.8)
May	1 (2.7)	30 (2.2)		206 (13.7)
June	0	29 (2.1)		133 (8.8)
July	2 (5.4)	79 (5.7)		110 (7.3)
August	11 (29.7)	190 (13.7)		133 (8.8)
September	6 (16.2)	372 (26.9)		171 (11.4)
October	8 (21.6)	280 (20.2)		171 (11.4)
November	3 (8.1)	182 (13.2)		148 (9.8)
December	3 (8.1)	89 (6.4)		87 (5.8)
Country				
England	24 (64.9)	888 (64.2)		858 (57.0)
Wales	1 (2.7)	188 (13.6)		246 (16.3)
Scotland	12 (32.4)	307 (22.2)	402 (26.7)	
Residence				
Government Office Region (England and Wales)				
Eastern	9 (24.3)	154 (11.1)	154 (10.2)	
East Midlands	3 (8.1)	252 (18.2)	129 (8.6)	
London	0	13 (0.9)	2 (0.1)	
North East	0	22 (1.6)	7 (0.5)	
North West	3 (8.1)	142 (10.3)	137 (9.1)	
South East	3 (8.1)	71 (5.1)	85 (5.6)	
South West	3 (8.1)	70 (5.1)	149 (9.9)	
Wales	1 (2.7)	188 (13.6)	246 (16.3)	
West Midlands	2 (5.4)	88 (6.4)	146 (9.7)	
Yorkshire and Humber	1 (2.7)	76 (5.5)	49 (3.3)	
Health Board (Scotland)				
Ayrshire and Arran	0	20 (1.4)	6 (0.4)	
Borders	0	3 (0.2)	29 (1.9)	
Dumfries and Galloway	1 (2.7)	17 (1.2)	50 (3.3)	
Fife	0	9 (0.7)	15 (1.0)	
Forth Valley	0	25 (1.8)	16 (1.1)	
Greater Glasgow and Clyde	3 (8.1)	55 (4.0)	52 (3.5)	
Grampian	4 (10.8)	49 (3.5)	81 (5.4)	
Highland	1 (2.7)	7 (0.5)	34 (2.3)	
Lanarkshire	0	23 (1.7)	12 (0.8)	
Lothian	3 (8.1)	53 (3.8)	45 (3.0)	
Orkney	0	2 (0.1)	8 (0.5)	
Shetland	0	0	0	
Tayside	0	44 (3.2)	54 (3.6)	
Western Isles	0	0	0	
